# Analysis of Key Material Parameters of Evaporator Wicks and Working Fluids for a Loop Heat Pipe Operating in the Temperature Range of 500–700 K

**DOI:** 10.3390/ma18122798

**Published:** 2025-06-13

**Authors:** Paweł Szymański, Piotr Radomski, Jae-Ho Jeon, Dariusz Mikielewicz

**Affiliations:** Institute of Energy, Faculty of Mechanical Engineering and Ship Technology, Gdańsk University of Technology, Gabriela Narutowicza St., 11/12, 80-233 Gdańsk, Poland; piotr.radomski@pg.edu.pl (P.R.); jae.ho.jeon@pg.edu.pl (J.-H.J.); dariusz.mikielewicz@pg.edu.pl (D.M.)

**Keywords:** loop heat pipe, capillary pressure, working fluid, ceramic wick, sintered metal wick

## Abstract

This study presents a preliminary evaluation of candidate wick material and working fluid for a flat-loop heat pipe (F-LHP) designed to operate within the temperature range of 500–700 K. The selection process considered key thermal and physical parameters, including thermal conductivity, chemical compatibility between wick and fluid, capillary pressure generation, pressure drop across the wick structure, and structural integrity at elevated temperatures. A range of metallic and ceramic wick materials, along with suitable high-temperature working fluids, were reviewed and compared based on performance metrics and practical availability. Special attention was given to oxidation and corrosion resistance, capillary performance, and thermal stability under elevated-temperature conditions. Nine different porous wicks with distinct materials and microstructures—differing in pore size, porosity, and permeability—were analyzed in combination with seven different working fluids. The analysis focused on determining which combinations generated the highest capillary pressure and which exhibited the lowest flow resistance due to external flow, thereby enhancing the LHP’s performance. Based on these results, the study identifies the most effective wick–fluid pairings for F-LHP applications, offering an optimal balance of thermal performance and long-term reliability. These findings provide a foundation for further experimental validation and the development of prototypes.

## 1. Introduction

Loop heat pipes (LHPs) are highly efficient, passive two-phase heat transfer devices that rely on the capillary action of a porous wick structure to circulate a working fluid without the use of mechanical pumps. Their capability to transport significant heat loads over long distances with high reliability and no moving parts makes them attractive for a wide range of thermal management applications, including aerospace systems, electronics cooling, and high-temperature industrial technologies. Among various LHP configurations, the flat-loop heat pipe (F-LHP) has attracted growing attention due to its compact design and suitability for integration with planar heat sources [1].

The performance and reliability of an F-LHP are critically dependent on two main components: the wick structure, which generates the capillary pressure necessary for fluid transport, and the working fluid, which undergoes phase change to absorb and release thermal energy. At elevated operating temperatures, particularly in the range of 500–700 K, the selection of appropriate materials becomes increasingly challenging. The wick must retain its structural integrity and capillary performance while resisting thermal degradation, corrosion, and chemical interaction with the working fluid. Likewise, the fluid must exhibit favorable thermophysical properties such as high latent heat of vaporization, good thermal stability, low viscosity, and chemical compatibility with the wick and structural materials of the LHP.

Despite extensive research on LHPs operating at or near room temperature, studies addressing high-temperature applications remain limited. However, this temperature range is becoming increasingly important in emerging technologies such as space systems, power electronics, and concentrated solar power, where reliable and efficient passive thermal control is crucial. A review of the literature reveals a noticeable gap in data related to LHP performance above 500 K, with only a few relevant studies conducted by Anderson et al. [2,3,4,5,6,7,8,9,10,11] and Faghri et al. [12,13]. To the best of the authors’ knowledge, currently only one research group, that of Werner et al. [14,15,16,17,18], is actively pursuing investigations in this area; however, their work is also still ongoing. This gap underscores the need for more focused investigations of suitable working fluids and wick materials capable of withstanding such demanding thermal environments. The extension of LHP operational ranges to elevated temperatures is strongly linked to the careful selection of both fluid and solid-phase materials, including their chemical and thermal compatibility as well as the structural design of the wick.

**Figure 1:**
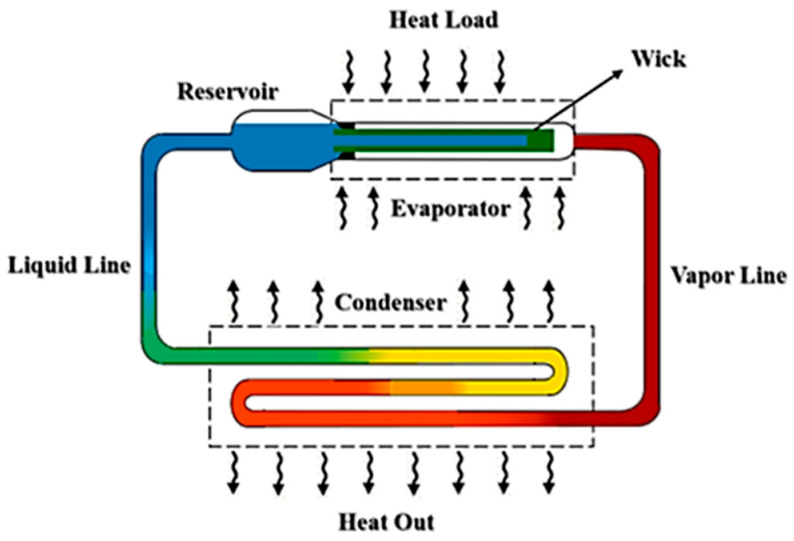
A general schematic of the LHP [19].

The operating principle of an LHP is based on a closed-loop circulation of the working fluid driven by capillary forces generated within a porous wick structure. An LHP typically comprises an evaporator, a compensation chamber (a.k.a. reservoir), a condenser, vapor and liquid transport lines, and a capillary wick. Heat input at the evaporator causes the liquid within the wick to evaporate at the interface adjacent to the vapor space. The capillary wick, saturated with liquid, not only provides the necessary driving force for fluid circulation but also serves as a barrier to vapor backflow, maintaining directional flow. The generated vapor travels through the vapor line toward the condenser, where it releases latent heat to an external heat sink and condenses. The resulting liquid is collected in the compensation chamber, which plays a critical role in regulating both the internal pressure and the liquid inventory within the system before being redistributed to the wick. This passive cycle enables continuous operation, and the capillary action ensures robust thermal performance even under microgravity or varying orientations. A schematic representation of the LHP configuration is shown in Figure 1.

The wick structure is vital to LHP function, as it creates the pressure differential necessary to overcome flow resistance and maintain fluid circulation. Key parameters influencing wick performance include porosity, permeability, average pore radius, and structural stability under thermal loading. A particular advantage of LHPs is that the meniscus within the wick dynamically adjusts to changes in heat load, decreasing its radius of curvature as the load increases to maximize capillary pressure. This pressure can be described by the Young–Laplace equation:

(1) $${\Delta P}_{cap}=\frac{2\cdot \sigma \cdot cos\theta }{r_{p}}$$

(2) $${\Delta P}_{max}={\Delta P}_{cap}\left(\theta =0\right)=\frac{2\cdot \sigma }{r_{p}}$$

where $r_{p}$ is the single pore radius, σ is the surface tension of the liquid, and θ is the contact angle between the liquid and the wick material. This capillary pressure must be sufficient to overcome the total pressure losses in the system, including those due to fluid flow through the wick, which are described by Darcy’s law:

(3) $$\Delta P_{wick}=\frac{l\cdot \mu_{f}}{\rho_{f}\cdot A_{f}\cdot K_{f}}\cdot {\dot{m}}_{f}$$

where $\mu_{f}$ is the dynamic viscosity of the fluid, $K_{f}$ is the permeability of the wick, *l* is a length of the wick, $A_{f}$ is the flow area to length ratio, $\rho_{f}$ is a density of the fluid and ${\dot{m}}_{f}$ is the fluid mass flow rate. The permeability of the wick is in turn a function of pore size and porosity, which can be approximated using the Carman–Kozeny equation [20,21]:

(4) $$K_{f}=\frac{{r_{p}}^{2}\cdot \varepsilon^{3}}{37.5\cdot {\left(1-\varepsilon \right)}^{2}}$$

where ε is the porosity of the wick. The physical properties of the working fluid can be found in a fluid databases, i.e., [22,23,24,25,26].

In addition to thermal and structural considerations, chemical compatibility between the working fluid and the wick material plays a crucial role, especially at elevated temperatures. Some combinations of materials and fluids may lead to chemical degradation, pore clogging, or corrosion, ultimately impairing the performance and lifespan of the device. For this reason, early-stage characterization of the chemical composition and purity of wick materials is essential during the design process.

This work aims to establish a preliminary foundation for the selection of suitable wick structures and working fluids in F-LHPs operating at elevated temperatures. This is a theoretical study, focused on analysis and evaluation rather than experimental validation. By identifying and evaluating key material properties—capillary pressure, pressure losses in the system related to permeability, thermal conductivity, and compatibility—this study seeks to support future experimental efforts and guide the development of high-temperature LHP prototypes for advanced thermal management applications.

## 2. Materials and Methods

### 2.1. Materials

As part of the conducted research, nine different wick structures were investigated, each fabricated from distinct materials and characterized by varying porosity and permeability. The studied wicks included sintered powder wicks made of SS316L stainless steel (AISI 316L/1.4404), with a temperature resistance up to 500 °C in oxidizing atmospheres. These wicks were custom-fabricated by the company Tridelta Siperm GmbH [27]. In addition, ceramic wicks composed of silicon carbide (SiC) were examined. One type incorporated high-temperature glass custom-fabricated by the Powloka company [28], while another consisted of mullite-bonded SiC, manufactured via uniaxial pressing of a powder mixture containing 80 wt% SiC and 20 wt% kaolin, followed by conventional sintering at 1500 °C for 2 h in air custom fabricated by Korea Institute of Materials Science [29].

**Figure 2:**
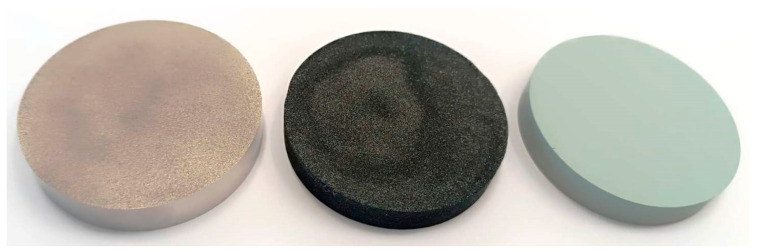
A photo of selected wicks made from (left to right) stainless steel, silicon carbide+glass, and silicon carbide + kaolin, respectively.

Table 1 specifies the investigated wicks, whereas Table 2 presents the thermal properties of the selected working fluids in this paper. Each wick had a cylindrical shape with dimensions of 60 mm in diameter and 100 mm in height. The photo in Figure 2 presents the external view of selected wicks.

### 2.2. Methods

To assess the applicability of the wicks and to reduce their number to the most effective candidates, their thermal resistance and effective thermal conductivity under the operational conditions of the working medium were also analyzed. It should be taken into account, however, that the accuracy of this evaluation was restricted by the irregular geometric structure of the sintered wick and the non-uniform deformation occurring during the sintering process. Hence, the models, which describe the effective thermal conductivity, tended to idealize the material as a continuous solid interacting with liquid spheres of varying diameters. In this work, the porosity of the wicks and their interaction with working fluids were incorporated into the wick’s thermal conductivity coefficient, $\kappa_{w}$, considering it as a function of the operating temperature, T, and expressed by [20]:

(5) $$\kappa_{w}=\kappa_{w}\left(T\right)=\kappa_{s}\left(T\right)\cdot \left(\frac{2+\frac{\kappa_{f}\left(T\right)}{\kappa_{s}\left(T\right)}-2\cdot \varepsilon \cdot \left(\frac{1-\kappa_{f}\left(T\right)}{\kappa_{s}\left(T\right)}\right)}{2+\frac{\kappa_{f}\left(T\right)}{\kappa_{s}\left(T\right)}+\varepsilon \cdot \left(\frac{1-\kappa_{f}\left(T\right)}{\kappa_{s}\left(T\right)}\right)}\right)$$

where $\kappa_{s}$ is the thermal conductivity coefficient of the solid of which the wick is made, $\kappa_{f}$ is the thermal conductivity coefficient of the working fluids at which the wick operates.

For LHP application, the effective thermal conductivity coefficient should be analyzed to reach its maximum perceptible value in order to intensify heat transfer and accelerate attainment of steady-state conditions. On the other hand, the operating pressure of the working fluid should be minimized, and the proper selection of materials, including the pipeline, is key to reduce the risk of system rupture due to excessive internal pressure, which may exceed the material’s shear or bending strength limits.

## 3. Results and Discussion

**Figure 3:**
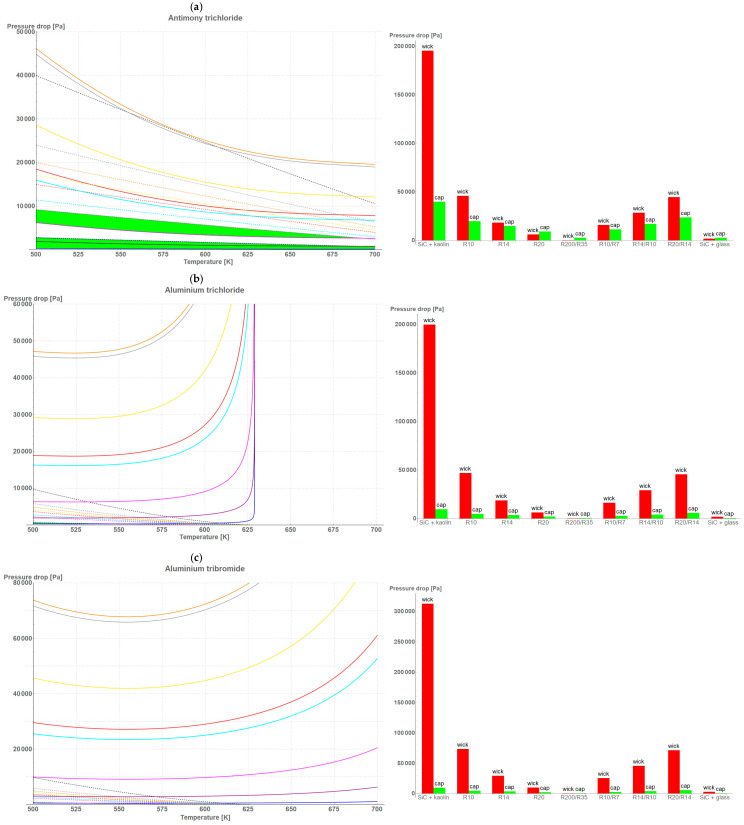

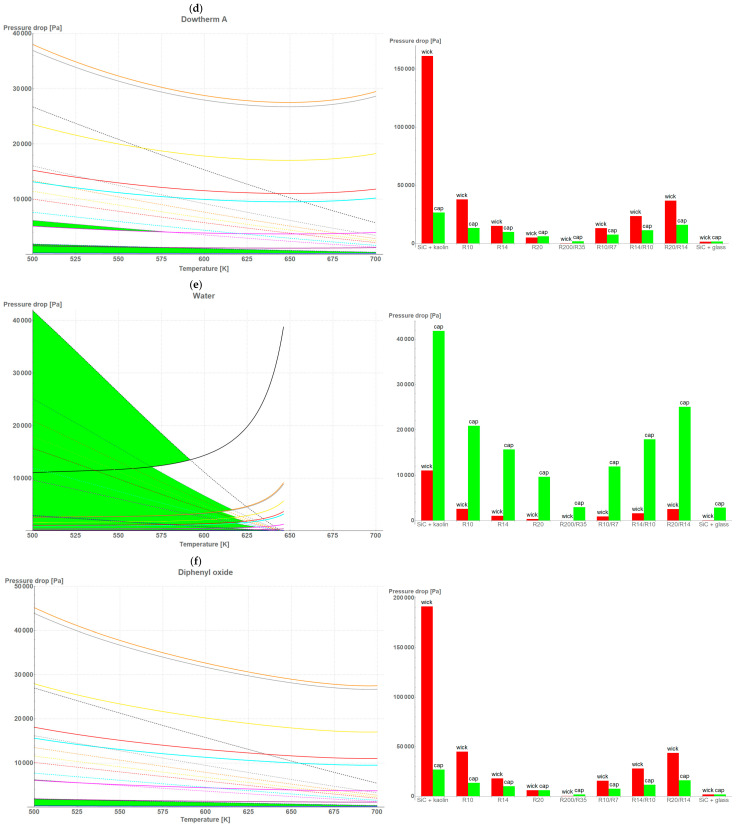

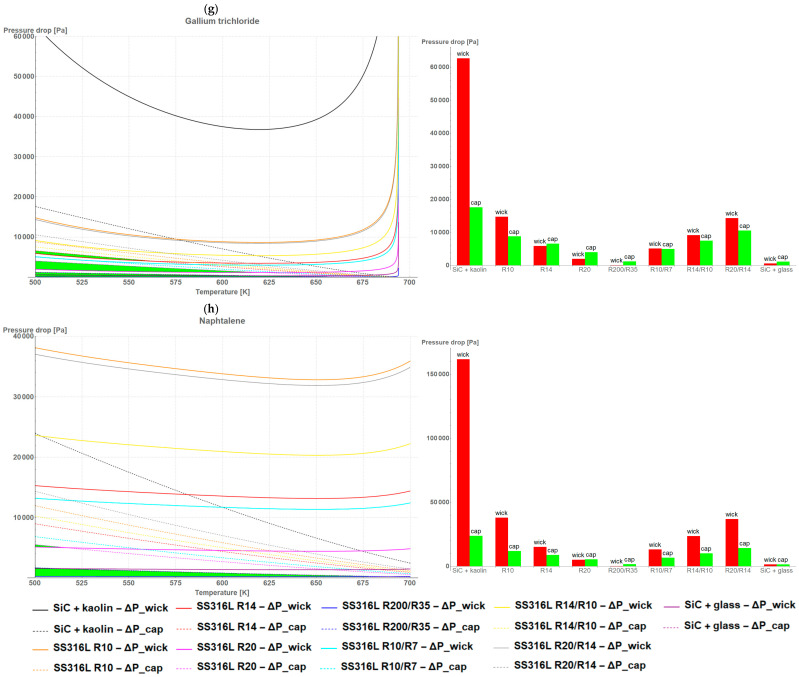
A comparative analysis of (**left**) wick performance in the 500–700 K temperature range, and (**right**) wicks’ applicability in maximum pressure drop for LHP working under a 100 W heat load for the following working fluids: (**a**) antimony trichloride; (**b**) aluminum trichloride; (**c**) aluminum tribromide; (**d**) Dowtherm A (**e**) water; (**f**) diphenyl oxide; (**g**) gallium trichloride; (**h**) naphthalene in 500–700 K temperature range. Areas marked by green color on **left** side highlight the performance temperature range of the selected wicks (${\Delta P}_{max}>{\Delta P}_{wick}$). Likewise, the wicks’ applicability on the charts on the **right**-hand side occurs when the green bar exceeds the red one.

Figure 3a–h reveal the capillary pressure, ${\Delta P}_{cap}$, and the pressure drop induced by a fluid flow in a wick, ${\Delta P}_{wick}$, respectively. As stated previously, the first analysis concerned the conditions at which ${\Delta P}_{max}$ was higher than ${\Delta P}_{wick}$. As may be noticed, the highest applicability was seen in the bi-porous high-porous wicks, such as the studied SS316L R200/R35. Pressure drops, however, were relatively small compared to those of the other wicks. Taking into account the wicks’ microstructures and the formula on pressure drops, it was found that:

- The higher the porosity of the wick, the wider the temperature range for the ${\Delta P}_{cap}>{\Delta P}_{wick}$ criterion and the lower the pressure drop value;
- The bigger the size of the pore, the higher the pressure drop maintaining the ${\Delta P}_{cap}>{\Delta P}_{wick}$ criterion;
- The bigger the area of the wick, the wider the temperature range for the ${\Delta P}_{cap}>{\Delta P}_{wick}$ criterion and the lower the pressure drop value;
- The thicker the wick, the wider the temperature range for the ${\Delta P}_{cap}>{\Delta P}_{wick}$ criterion and the lower the pressure drop value.

In case of working fluids, water stood out from the crowd; however, temperatures were near the critical value where vapor pressure drastically increased. A similar situation may be observed for AlCl_3_ and GaCl_3_.

**Figure 4:**
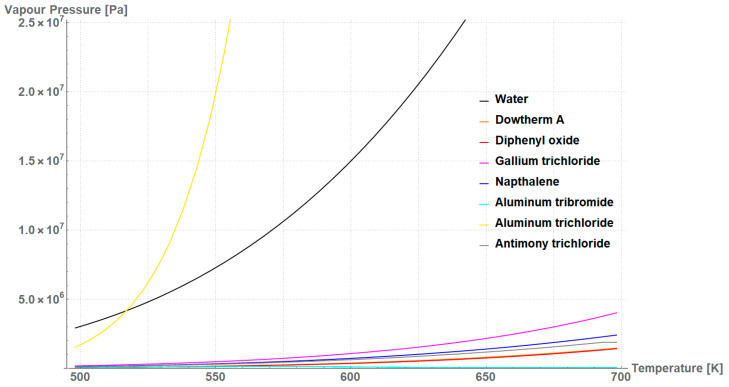
Vapor pressure of the considered working fluids. Notably, the difference in values between ‘Dowtherm A’ and ‘diphenyl oxide’, represented by the orange and red lines respectively, leads to an overlap of the lines in the figure.

Figure 4 presents the vapor pressure that expressed the functionality for the studied working fluids. Although water and AlCl_3_ exhibited the highest potential to be used for wick evaporators, the vapor pressure in the 500–700 K temperature range contributed to increase the risk of system rupture, which eliminated these candidates for further analysis. Table 3 highlights the comparative values for the SS316L R200/R35 wick at 500 K, where water’s applicability was still justified, and at 700 K—above the critical temperature of water. As a key parameter that eliminated working fluids in further analysis, the operating temperature, T, needed to be less than the critical temperature of the working fluid, $T_{crit}$ and the potential of evaporation had to be more than or equal to 1. The applicability assessment was based on an equally scored comparison (0.25 each) of criteria of the working fluids, which were: vapor pressure, which ranged from 2.9216 MPa, the maximum vapor pressure of water, 0; safety, which ranged from 0 to 12 (according to fire diamond NFPA 704); performance, which ranged from 1 to the maximum value per each operating temperature; and price, ranging from 0 to the most expensive product.

According to the results in Table 3, while water’s applicability at medium-elevated operating temperatures was indisputable, diphenyl oxide and Dowtherm A were promising substitutes at 700 K, because their properties enabled them to be used for evaporator wicks instead of water, which failed due to the critical temperature. According to the data in literature, the considered working fluids exhibit similar and low thermal conductivity, between 0.1 and 0.6 W/(m⋅K), whereas the highest value is assigned to water. Since the differences between fluids are minimal, the price and availability on the market differentiated the applicability of working fluids. In this work, vapor pressure, safety, performance, and price possessed an equal weight. In reality, customer can decide which parameter criterion can be emphasized more. Dowtherm A beat diphenyl oxide by almost nine times, taking into account only the price of the product. However, when the criteria were balanced, both Dowtherm A and diphenyl oxide were considered to be the best working fluids that substituted for water as an evaporator wick for LHP applications. It should be noted that although this analysis was conducted for the SS316L R200/R35 wick, this wick was an example and it was selected due to its broad applicability with a wide range of working fluids in LHP applications. Analysis of other structures and materials can be undertaken similarly.

## 4. Conclusions

This study provides a comprehensive analysis of wick structures and high-temperature working fluids for F-LHP application operating in the 500–700 K temperature range. By evaluating nine porous wick types and seven candidate fluids, the investigations focused on key performance parameters—capillary pressure, pressure drop in a wick, thermal conductivity, and vapor pressure of the working fluid.

Among the studied wicks, bi-porous stainless steel wicks with high porosity and large pore diameters, such as SS316L R200/R35, exhibited the most favorable characteristics, offering high capillary pressure with relatively low hydraulic pressure drop as a result of fluid flow. These aspects ensure good wick performance while maintaining the ${\Delta P}_{cap}>{\Delta P}_{wick}$ criterion in a wide temperature range, which is crucial for the LHP applications.

In terms of working fluids, while water and AlCl_3_ showed superior thermal properties, their high vapor pressures at elevated temperatures can bring a significant risk of rupture, and they were considered not to be suitable for long-term applications in high-temperature systems. Conversely, Dowtherm A and diphenyl oxide emerged as the promising alternatives due to their low vapor pressures at similar thermal stability compared to the rest of the working fluids. Dowtherm A, in particular, can be treated as a well-balanced candidate that would effectively substitute water in the temperature range 500–700 K taking into account the economic factors. Future studies should focus on experimental validation of the identified wicks and working fluids to confirm their performance.

## Figures and Tables

**Table 1:** Wicks’ specification [27,28,29].

Wick	Materials	Shear Strength	Diameter	Thickness	Porosity	Pore Diameter
Ceramic	Silicon carbide + glass	76 MPa	0.06 m	0.1 m	23.5%	44 μm
Ceramic	Silicon carbide + kaolin	76 MPa	0.06 m	0.1 m	35.0%	3.0 μm
SS316L R10	Stainless steel	240 MPa	0.06 m	0.1 m	35.5%	6.0 μm
SS316L R14	Stainless steel	210 MPa	0.06 m	0.1 m	38.5%	8.0 μm
SS316L R20	Stainless steel	180 MPa	0.06 m	0.1 m	39.6%	13.0 μm
SS316L R200/R35	Stainless steel	80 MPa	0.06 m	0.1 m	45.5%	42.5 μm
SS316L R10/R7	Stainless steel	240 MPa	0.06 m	0.1 m	35.0%	5.0 μm
SS316L R14/R10	Stainless steel	210 MPa	0.06 m	0.1 m	37.0%	7.0 μm
SS316L R20/R14	Stainless steel	180 MPa	0.06 m	0.1 m	39.0%	10.5 μm

**Table 2:** Thermal properties of the working fluids considered in this paper [22,23,24,25,26].

Working Fluid	Chemical Formula	Melting Point	Boiling Point ^1^	Critical Temperature	Critical Pressure
Water	H_2_O	273.15 K	373.15 K	647.10 K	22.12 MPa
Aluminum tribromide	AlBr_3_	370.65 K/366.15 K ^2^	528.15 K	763.15 K	2.89 MPa
Aluminum trichloride	AlCl_3_	453.15 K/373.15 K ^2^	455.85 K	625.75 K	2.60 MPa
Antimony trichloride	SbCl_3_	346.55 K	496.65 K	794.15 K	4.82 MPa
Gallium trichloride	GaCl_3_	351.05 K/317.55 K ^3^	474.15 K	694.15 K	6.11 MPa
Diphenyl oxide	(C_6_H_5_)_2_O	298.15 K	531.70 K	767.15 K	3.29 MPa
Naphthalene	C_10_H_8_	351.35 K	491.12 K	748.35 K	4.11 MPa
Dowtherm A	(C_12_H_10_)/(C_12_H_10_O) ^4^	285.15 K	530.15 K	770.15 K	3.13 MPa

^1^ At atmospheric pressure, $p_{atm}=1013\;hPa$; ^2^ anhydrous/hexahydrate; ^3^ anhydrous/monohydrate; ^4^ eutectic mixture made of two chemical compounds.

**Table 3:** Comparative assessment of working fluids as a water substitute for 500 K and 700 K for the SS316L R200/R35 wick. A similar procedure can be followed for other wicks. Table cells marked in red indicate that basic criteria were not met.

Working Fluid	$T\;<\;T_{crit}$	Vapor Pressure (MPa)	Safety ^1^	Potential of Evaporation ^2^	Price (USD) ^3^ for 100 g	Assessment (0–1)
T=500 K						
Water	**✓**	2.9216	0	167.4993	5.66 ^4^	0.7497
Aluminum tribromide	**✓**	1.5366	5	2.1631	517.02	0.4890
Aluminum trichloride	**✓**	0.046335	5	1.3823	1851.06	0.5458
Antimony trichloride	**✓**	0.10325	3	9.0409	906.91	0.6434
Gallium trichloride	**✓**	0.17512	4	12.4307	4789.00	0.4189
Diphenyl oxide	**✓**	0.046893	3	6.2428	85.11	0.6869
Naphthalene	**✓**	0.11891	4	6.5514	185.11	0.6552
Dowtherm A	**✓**	0.049701	3	7.3383	21.22	0.6917
*T* = 700 K						
Water	**X**	-	0	-	5.66 ^4^	0.0000
Aluminum tribromide	**X**	-	5	-	517.02	0.0000
Aluminum trichloride	**X**	-	5	-	1851.06	0.0000
Antimony trichloride	**✓**	1.88546	3	5.6605	906.91	0.5262
Gallium trichloride	**X**	-	4	-	4789.00	0.0000
Diphenyl oxide	**✓**	1.4198	3	2.0564	85.11	0.6332
Naphthalene	**✓**	2.4087	4	0.7158	185.11	0.0000
Dowtherm A	**✓**	1.4580	3	2.0248	21.22	0.6458

^1^ Sum of numerals, according to NFPA 704 (0—totally safe; 12—totally hazardous); ^2^ expressed by the ratio ${\Delta P}_{max}/{\Delta P}_{wick}$; ^3^ price and currency rate on 5 May 2025; data are taken from https://www.sigmaaldrich.com/, accessed on 29 April 2025; ^4^ deionized water.

## Data Availability

The original contributions presented in this study are included in the article. Further inquiries can be directed to the corresponding author.

## Abbreviations

The following abbreviations are used in this manuscript:

cap	capillary
crit	critical
F-LHP	flat-loop heat pipe
LHP	loop heat pipe
max	maximum
SS316L	type 316L stainless steel
